# Modeling secondary level of HIV contact tracing: its impact on HIV intervention in Cuba

**DOI:** 10.1186/1471-2334-10-194

**Published:** 2010-07-01

**Authors:** Ying-Hen Hsieh, Yun-Shih Wang, Hector de Arazoza, Rachid Lounes

**Affiliations:** 1 Department of Public Health and Institute of Biostatistics, China Medical University, Taichung, Taiwan 404; 2 Department of Applied Mathematics, National Chung Hsing University, Taichung, Taiwan 402; 3 Department of Mathematics, University of La Habana, Havana, Cuba; 4 Laboratoire MAP5, Université Paris Descartes, UMR-CNRS 8145, Paris, France

## Abstract

**Background:**

Universal HIV testing/treatment program has currently been suggested and debated as a useful strategy for elimination of HIV epidemic in Africa, although not without practical issues regarding the costs and feasibility of a fully implemented program.

**Methods:**

A mathematical model is proposed which considers two levels of detection of HIV-infectives through contact tracing of known infectives in addition to detections through other means such as random screening. Simulations based on Cuban contact tracing data were performed to ascertain the potential impact of the different levels of contact tracing.

**Results:**

Simulation studies illustrate that: (1) contact tracing is an important intervention measure which, while less effective than random screening, is perhaps less costly and hence ideal for large-scale intervention programs in developing countries with less resources; (2) the secondary level of contact tracing could significantly change the basic disease transmission dynamics, depending on the parameter values; (3) the prevalence of the epidemic at the time of implementation of contact tracing program might be a crucial factor in determining whether the measure will be effective in preventing disease infections and its eventual eradication.

**Conclusions:**

Our results indicate that contact tracing for detection of HIV infectives could be suitably used to remedy inadequacies in a universal HIV testing program when designing timely and effective intervention measures.

## Background

A recent modeling study [1] on universal HIV screening followed by immediate antiretroviral treatment (ART) for those tested positive concludes that, assuming that the infectiousness of those treated fell to 1% of their infectiousness before treatment, this strategy could have a major effect on HIV/AIDS epidemic. However, as noted in that article, screening everyone periodically, whether voluntary or not, is not feasible in practice. Moreover, the costs of periodic universal testing program might be prohibitively expensive, especially in the developing countries.

The HIV/AIDS epidemic in Cuba has had significantly low prevalence compared to its neighboring countries in the Caribbean Basin, which has the second highest rate of HIV/AIDS in the world after sub-Saharan Africa [2]. The Joint United Nations Program on HIV/AIDS (UNAIDS) report on the global HIV/AIDS epidemic for 2007 indicates that the region's HIV epidemics are in general highly prevalent and driven primarily by heterosexual intercourse. In contrast, estimates for Cuba indicate an adult HIV prevalence of 0.1%, out of 6.1 million persons in the age group 15-49 [3]. Moreover, Men having sex with men (MSM) continue to be the main high prevalence group in Cuba, with most of the HIV-positive adults being men (77%) and most of the detected HIV-positive men being reported as MSM (85.1%), while most of the HIV-positive women reported having had sex with MSM [4].

The continued low HIV prevalence in Cuba has its root in its distinct social factors as well as in its unique intervention program. The Cuban HIV/AIDS programme included a system to detect HIV cases from several sources. Some of these sources were used at the beginning of the programme, others were introduced later and some have been discontinued. Since 1993, this detection system has focused on 6 major sources: blood donors; persons treated for other sexually transmitted infections; persons admitted to hospital with suspected HIV infection or subject to specific procedures like dialysis; persons volunteering to be tested; persons whose general practitioner has recommended HIV testing; and sexual partner tracing. Other less important sources include testing of all pregnant women and prison inmates. One of the major reasons for the low HIV prevalence in Cuba compared to its neighbors in the Caribbean region is its sexual contact tracing program, also known as the Partner Notification Program. Since 1986, a person testing HIV-positive in Cuba is interviewed by health workers using a non-anonymous structured questionnaire. They are invited to give names and contact details of their sexual partners of the past two years. These partners are then traced and a recommendation for voluntary HIV testing is made with follow-up testing for up to one year [5].

Modeling of Cuba HIV has been carried out in recent years, some focusing mainly on underreporting [6-8] and others on estimation of HIV-infected population sizes using Generalized Removal Model for Open Population or GERMO [5,9]. Several studies also focused on modeling the contact tracing program in Cuba includes [8,10-13], mainly with the Cuba HIV/AIDS epidemic as the background due to the availability of detailed HIV case and contact tracing data.

Sexual transmission is the primary mode of infection in Cuba, in particular that of MSM group [4]. Hence seeking out sexual contact networks through contact tracing is a vital aspect of intervention and control of the HIV epidemic in Cuba. In the previous modeling work on contact tracing, a detection rate by contact tracing is assigned for all detected individuals, thus ignoring the difference between the first level of traced contacts (i.e., those who were traced earliest as members of a newly discovered network of direct sexual contacts of an infective detected through means other than contact tracing) and the succeeding levels of traced contacts (i.e., those in the network who were traced consequently as subsequent contacts of traced contacts in a network). For example, those infectives traced and diagnosed as reported contacts of the first level of traced contacts belong to the second level, and those traced as reported contacts of the second level of traced contacts belong to the third level, and so on. In this work, we will extend our previous model to include a second level of contact tracing, that is, those who were detected as sexual contacts of those who were the earliest detections through contact tracing as contacts of those who were detected through means other than contact tracing.

**Figure 1:**
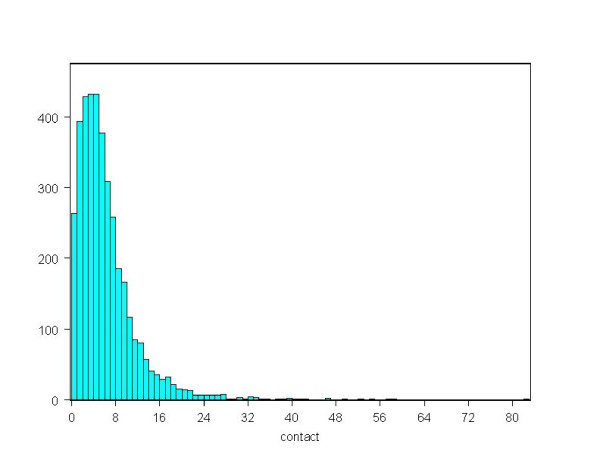
**Histogram of the numbers of contacts declared by the 4091 HIV-infected persons in the 1986-2001 Cuban contact tracing data**. X-axis is the number of contacts and y-axis is the number of detected HIV-positive individuals with the given number of contacts.

The main source for this extension is the detailed study of the 1986-2001 Cuban contact tracing data of 4091 HIV-infected persons detected by the beginning of 2002. Of those detected, 6.81% gave no contacts or refused to give any contact information and the average number of contacts is 5.84 with 95% CI: (5.66, 6.02). Fig. 1 gives a histogram of the number of contacts declared.

**Figure 2:**
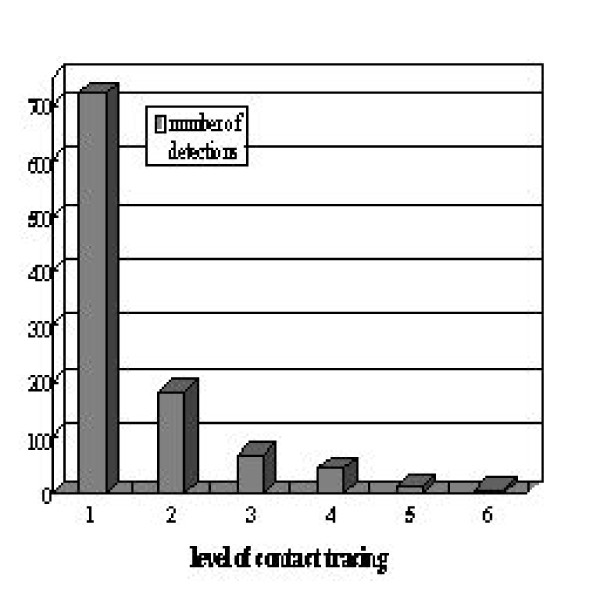
**Distribution of number of detections for levels 1-6 of contact tracing in the 1986-2001 Cuban contact tracing data**.

The data also revealed that 1221 (30%) were detected through contact tracing, i.e., they were tested positive after being identified as contacts of HIV-infected persons. There were some large sexual contact networks with the largest three networks having 87, 115, and 158 detected members, respectively. However, there were only 24 networks with 10 or more detected members totaling 664 (54%) known infectives, thus indicating that most networks are very limited in size, although they combine to contain almost half of the 4091 infected individuals,. Of the 1221 persons detected through contact tracing, 1026 can be traced to a contact network. Furthermore, of those known to belong to a network, 719 (70%) were identified as contacts of person who were detected by means other than contact tracing. In other words, these 719 persons are detected through the first level of contact tracing. Moreover, the number detected through subsequent contact tracing drop off dramatically after the initial level of contact tracing, to 181 (18%) at second level of contact tracing (those identified as contacts of those 719 first level detections) and 69 (6.7%) at the third level. The complete distribution of number of detection by the first 6 levels of contact tracing is given in Fig. 2.

Fig. 2 gives evidence that the effectiveness of contact tracing decreases sharply as tracing into the tertiary levels is being conducted, most likely due to the gradual exhaustion of the undetected members in the sexual contact network to which the detected infectives belong, and also due to the overlapping between infection chains or sexual networks. Moreover, the information it provides becomes redundant and obsolete as the contact tracing program continues, since large-scale contact tracing is a time-consuming process and requires personnel that must be exclusively dedicates to this task, especially in large population centers. Therefore, we wish to ascertain the true effectiveness of the contact tracing program, by gauging the impact of secondary and perhaps tertiary levels of contact tracing.

Early mathematical models of screening and treatment for HIV include [14-17]. Ample work on modeling sexual partnership network had been carried out in recent years, e.g., [18-25]. However, these models were mostly proposed to construct sexual partnership (concurrent or otherwise) networks.

In the context of the HIV epidemic in Cuba, contact tracing exhibit only partial results of the partnership networks, filtered through non-random detection of infected individuals, recall bias of these infected individuals, tracing and testing of the contacts, timing of the tracing and testing, etc. Moreover, the Cuban contact tracing data contains only those tested positive. Therefore, to use the approaches proposed in the above models would lead to an even more complicated (compartmental or individual-based) model which requires additional data on sexual network which is rarely available, with additional assumptions and further uncertainties in the results obtained. Hence, in this work, we will consider a simple compartmental model which enables us to focus on the secondary level of contact tracing and its relative impact.

## Methods

### The Model

**Figure 3:**
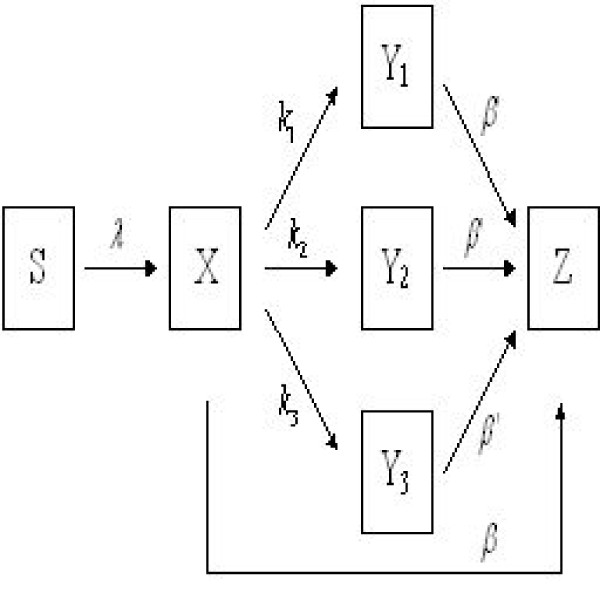
**Model flow diagram**. The mortality rates of all compartments are omitted for sake of brevity.

We consider a compartmental model as diagramed in Fig 3. The model variables are given below:

S(t) is the susceptible population.

X(t) is the number of HIV-infected persons that do not know they are infected at time t.

Y_1_(t) is the number of HIV-infected persons that are known by the health authority to be infected at time t and were detected in a random type search.

Y_2_(t) is the number of HIV-infected persons that are known by the health authority to be infected at time t and were detected at the first level of contact tracing as past contacts (or sexual partners) of Y_1_.

Y_3_(t) is the number of HIV-infected persons that are known by the health authority to be infected at time t and were detected at the second level of contact tracing as past contacts of Y_2_.

Z(t) is the number of persons with AIDS at time t.

The model parameters are listed below:

Λ: the constant recruitment rate into the susceptible population.

*λ*: the rate of the new HIV-infected persons are infected by X(t).

*k*_1_: the rate at which the unknown HIV-infected persons are detected by the system, independently of other HIV-positive persons (through "random" screening).

*k*_2_: the rate at which the unknown HIV-infected persons are detected by the system through contact of Y_1_.

*k*_3_: the rate at which the unknown HIV-infected persons are detected by the system through contact of Y_2_.

*β*: the rate at which the undetected HIV-positive persons develop AIDS.

*β'*: the rate at which the detected HIV-positive persons develop AIDS.

*μ*: mortality rate of the sexually active population.

*μ'*: mortality rate of the population with AIDS.

Following previous modeling work (e.g. [7,8,12,13]), we assume a constant recruitment rate *λ *of the new infectives, infected by the infectives who did not know that they were infected. The assumption of linear rate of recruitment of new HIV-infected persons infected by X(t), *λ *X, is based on our background setting of Cuba, where the prevalence is low. We also assume linear detection rates by contact tracing, k_2 _and k_3_, where a certain fraction of previous contacts of a known infective is successively traced and tested to be HIV-positive. A class of linear and nonlinear functions for detection via contact tracing was considered in [13], in which the nonlinear detection by contact tracing term used in this model is the one found to give best fit for Cuban HIV data. Moreover, estimates for k_2 _were also obtained in [7].

We also assume that the infection by detected infectives is negligible when compared with the infection rate by the unknown infectives, since it was estimated in [12], using Cuban HIV/AIDS data between 1986-2000, that the infection rate by the known infectives in Cuba is only around 5.79% (SD = 3.55) of that of unknown infectives. We also ignore the contact tracing at the third level and after, since the tertiary levels of detections are small when compared with that of the second level.

The model dynamics is described by the following system, with the time unit in years:(2.1)

Note that System (2.1) is well-defined in the 4-dimensional positive orthant of the XY_1_Y_2_Y_3_-space. For a theoretical analysis for models of this type near the origin, see [26].

## Results

### Steady states and threshold conditions

We consider only four equations in system (2.1) which are relevant to our analysis, namely, the second to the fifth equations, and let *x *= *X*/(*X *+ *Y*_1 _+ *Y*_2 _+ *Y*_3_), *y*_*i *_= *Y*_*i *_/(*X *+ *Y*_1 _+ *Y*_2 _+ *Y*_3_), *i *= 1, 2, 3, be the respective fractions of *X *and *Y*_*i *_in the infective population. Using the fact that *x *+ *y*_1 _+ *y*_2 _+ *y*_3 _= 1, we obtain the 3-dimensional dynamical system:(3.1)

System (3.1) has several dynamic steady states (also known as equilibrium points), each with its corresponding threshold condition for stability.

1. Trivial steady state: *E*_0 _= (0, 0, 0)

2. Boundary steady states: *E*_*B*1 _= (0, *r*_2_,0), *E*_*B*2 _= (0, 0, *r*_3_) and  where , and  are nonzero numbers between 0 and 1, and their values depend on the initial fractions of the solution trajectories at time t = 0, i.e.,(*x*(0), *y*_2_(0), *y*_3_(0)).

(i) *E*_*B*1 _= (0, *r*_2_,0), where 0 <*r*_2 _≤ 1.

(ii) *E*_*B*2 _= (0, 0, *r*_3_), where 0 <*r*_3 _≤ 1.

(iii) , where  and .

3. Positive endemic steady state: , .

Note that since we have assumed that the infections by the detected infectives are negligible, the endemic steady state E* is the only endemic steady state where some infectives have not been detected and continue to cause new infections in the community. The threshold condition for each of the steady states is as follows:

(a) If *R*_0 _< 1, then *E*_0 _= (0, 0, 0) is locally asymptotically stable for (2.1) where *R*_0 _= (*λ *- *β *+ *β'*)/(*k*_1 _+ *k*_2_).

(b) If *R*_*B*1 _< 1, then *E*_*B*1 _= (0, *r*_2_,0) is locally asymptotically stable for (2.1) where *R*_*B*1 _= (*λ *- *β *+ *β'*)/[*k*_1 _+ *k*_2 _- (*k*_2 _- *k*_3_)*r*_2_] and 0 <*r*_2 _≤ 1.

(c) If *R*_*B*2 _< 1, then *E*_*B*2 _= (0, 0, *r*_3_) is locally asymptotically stable for (2.1) where *R*_*B*2 _= (*λ *- *β *+ *β'*)/(*k*_1 _+ *k*_2 _- *k*_2_*r*_3_) and 0 <*r*_3 _≤ 1.

(d) If *R*_*B*3 _< 1, then  is locally asymptotically stable for (2.1) where ,  and .

(e) If R* < 1, then  is locally asymptotically stable for (2.1) where

The above results can be easily derived by making use of Jacobian matrix and hence is omitted.

### Simulations Studies

We will now give some numerical examples to illustrate our results. Recall that all equilibria except the endemic steady state E* listed in the previous section are essentially disease free, since the fraction of undetected HIV-infectives, where individuals in all other infective classes must come from, is 0. We also note that the sizes of threshold parameters, *R*_*B*1_, *R*_*B*2_, *R*_*B*3_, and R*, depend on , and , and subsequently on the initial fractions used for the simulations.

**Figure 4:**
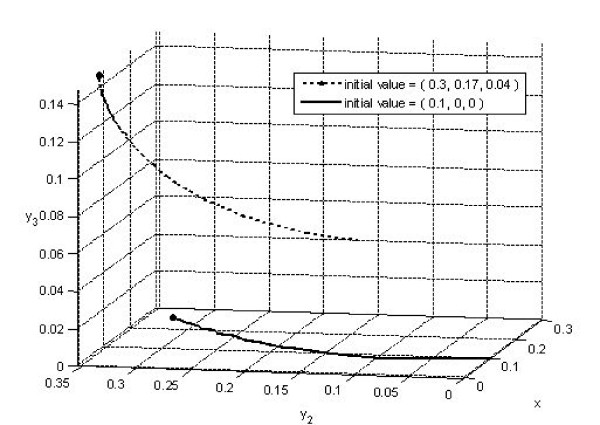
Simulation of the case (*k*_1_, *k*_2_, *k*_3_) = (0.25,0.5,0.25) for rows 3 and 4 of Table 1, where the dotted blue trajectory approaches the endemic steady state E* while solid green trajectory approaches DFE *E*_*B*3_.

For our numerical simulations utilizing Phaser 2.1 scientific software for simulating dynamical systems, we use the following parameter values estimated directly from the 1987-2000 Cuban demographic and HIV/AIDS data (see [7,10,13]): *μ *= 0.0053, *λ *= 0.5744, *β *= 0.1135, *β' *= 0.1350. Moreover, from the results of [13] we let *k*_1 _= 0.25 and *k*_2 _= 0.25. For our first simulation, we also assume that the detection rate for secondary level of contact tracing is the same as the first, i.e., *k*_3 _= 0.25, and the initial fractions are set at (*x*(0), *y*_2_(0), *y*_3_(0)) = (0.3,0.17,0.04) in keeping with the 1986-2001 Cuban HIV/AIDS and contact tracing data as described earlier. Hence, our first simulation uses post-2001 years in Cuba as the background setting. The resulting threshold parameters and the limiting behavior are summarized in row 1 of Table 1, where all threshold parameters except R* are greater than 1, hence the solutions approach the endemic steady state E*. This seems to reflect the actual HIV epidemic in Cuba since 2002, where slow but steady growth in HIV case number has been observed (Fig. 2, [4]).

**Table 1:** Simulations for model with first and second levels of contract tracing

Parameters values			Reproduction numbers					Limiting steady state
***k*_1_**	***k*_2_**	***k*_3_**	***R*_0_**	***R*_*B*1_**	***R*_*B*2_**	***R*_*B*3_**	R*	

0.25	0.25	0.25	1.19	1.19	1.24	1.24	0.67	endemic steady state E*
0.375	0.25	0.25	0.95	0.95	0.996	0.996	1.004	DFE *E*_*B*3_
0.25	0.5	0.25	0.79	0.90	0.88	1.01	0.92	endemic steady state E*

**0.25**	**0.5**	**0.25**	**0.79**	**0.87**	**0.81**	**0.89**	**0.79**	**DFE ***E*_*B*3_

The initial fractions for the first 3 rows are (*x*(0), *y*_2_(0), *y*_3_(0)) = (0.3,0.17,0.04), initial fractions for the last row in **bold **is (*x*(0), *y*_2_(0), *y*_3_(0)) = (0.1, 0, 0).

If we increase detection by means other than contact tracing (and in the process increase the number of infectives whose contacts are to be traced) by 50%, all other reproduction numbers are less than 1 with the exception R* which is greater than 1, thus guaranteeing that solutions will become disease-free (second row of Table 1). In this instance, the solution approaches *E*_*B*3_, the steady state with nonzero fractions of infectives traced by first and second level contact tracing. However, if instead we double the first level contact tracing rate, only *R*_*B*3 _is greater than 1 and our solution goes to the endemic equilibrium (third row, Table 1). Hence increasing detection through means other than contact tracing might be more effective, although perhaps also more costly.

To further illustrate the effect of different initial fractions on the asymptotic behavior of the system, the fourth row (in bold) in Table 1 has the same parameters as row 3, but the initial fractions had been changed to (*x*(0), *y*_2_(0), *y*_3_(0)) = (0.1, 0, 0). That is, *x*(0) = 0.1 and *y*_1_(0) = 0.9, or 90% of the infective population had been detected through means other than contact tracing. In contrast to the case in row 3 where *R*_*B*3 _was greater than 1 and the trajectory approached the endemic steady state E*, here we have *R*_*B*3 _less than 1 and the trajectory approaches the DFE *E*_*B*3 _on the boundary of the positive octant (see the 3-d graph in Fig. 4).

**Figure 5:**
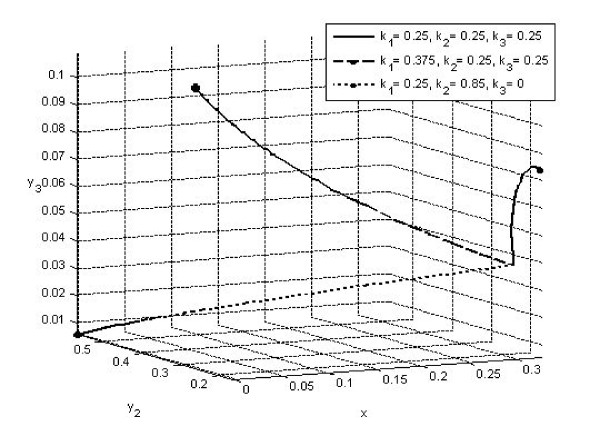
Simulation for the cases of row 1 (solid blue trajectory approaching E*) and row 2 (dash green trajectory approaching *E*_*B*3 _) in Table 1, and row 2 (dotted red trajectory approaching *E*_*B*1_) in Table 2, where initial fractions for all three trajectories are (*x*(0), *y*_2_(0), *y*_3_(0)) = (0.3,0.17,0.04).

Next, to highlight the impact of second level detection, we let *k*_3 _= 0, i.e., we suppose secondary contact tracing is abandoned at time t = 0, and subsequently obtain the simulation results in Table 2. Note that since there is no secondary contact tracing, *R*_*B*2 _and *R*_*B*3 _does not apply. First row shows that if only secondary contact tracing is not carried out, the solutions will still go to the stable endemic steady state, albeit with a higher percentage of undetected infectives (x* = 0.404 compared to 0.331 for row 1 of Table 1). Moreover, a slightly higher first level contact tracing rate will not alter the dynamics significantly (row 1, Table 2). Only when the first level contact tracing rate is drastically increased (more than tripled in row 2, Table 2), will the solution go to disease free. To illustrate, Fig. 5 provides simulations for the cases of row 1 (solid blue trajectory approaching E*) and row 2 (dash green trajectory approaching *E*_*B*3_) in Table 1, and row 2 (dotted red trajectory approaching *E*_*B*1_) in Table 2. The initial fractions for all three trajectories are (*x*(0), *y*_2_(0), *y*_3_(0)) = (0.3,0.17,0.04).

**Table 2:** Simulations for model with first level of contact tracing only after initial time t = 0

Parameters values			Reproduction numbers					Limiting steady state
***k*_1_**	***k*_2_**	***k*_3_**	***R*_0_**	***R*_*B*1_**	***R*_*B*2_**	***R*_*B*3_**	R*	

0.25	0.25	0	1.19	1.31	NA	NA	0.60	endemic steady state E*
0.25	0.85	0	0.54	0.99	NA	NA	1.02	DFE *E*_*B*1_

The initial fractions are (*x*(0), *y*_2_(0), *y*_3_(0)) = (0.3,0.17,0.04).

For the last set of simulations, we assume that contact tracing is abandoned after t = 0, i.e., k_2 _= k_3 _= 0, in order to ascertain the impact of contact tracing. The results are given in Table 3, where the same detection rate through means other than contact tracing would yield a large value of *R*_0 _= 2.38 and the solutions approach the endemic steady state (row 1). This detection rate would need to be increased substantially (more than two-fold) to 0.6 for lower *R*_0 _to be barely under 1 at 0.9932, but low enough for the solution to approach DFE at *E*_*B*3 _(row 2).

**Table 3:** Simulations for model without contract tracing

Parameters values			Reproduction numbers					Limiting steady state
***k*_1_**	***k*_2_**	***k*_3_**	***R*_0_**	***R*_*B*1_**	***R*_*B*2_**	***R*_*B*3_**	R*	

0.25	0	0	2.38	NA	NA	NA	NA	endemic steady state
0.6	0	0	0.99	NA	NA	NA	NA	DFE *E*_*B*3_

The initial fractions are (*x*(0), *y*_2_(0), *y*_3_(0)) = (0.3,0.17,0.04).

## Discussion and Conclusions

### Our results indicate the following

1. Increasing the efficiency of contact tracing (higher contact tracing rates k_2 _and k_3_) is less effective than other means of detection (e.g., random screening), but is perhaps less costly and more cost-effective, and hence is ideal for large-scale intervention programs in developing countries with limited resources.

2. Without contact tracing (k_2 _= k_3 _= 0), detection by other means must be increased substantially (more than two-fold in Table 3) to lower *R*_0 _to less than 1.

3. If the second level contact tracing is not carried out (k_3 _= 0), the proportion of undetected infectives x* will increase by about 22% (from 0.331 to 0.404) in our simulation. Moreover, drastic increase (by more than three-fold) in the first level contact tracing rate is need for the system to become disease free.

4. The results in Fig. 4 indicate that if random screening had been highly effective (90% in our simulation), implementation of contact tracing program could help to eradicate the disease in the sense that the endemic fractions (*y*_1_, *y*_2_, and *y*_3_) will go to 0 in time. However, if the random screening does not detect a sufficiently high percentage of infectives, the implementation of the same program will not prevent the disease from becoming endemic.

5. In terms of dynamics, the model with only one level of contact tracing (Hsieh *et al*. 2005) exhibits very simple dynamics where all solutions either approach the DFE or the endemic equilibrium. When secondary level of contact tracing is included, bistability might occur where the solution could approach either the endemic steady state or any of the DFEs (*E*_0 _or any one of the disease-free boundary steady states), as illustrated in rows 3 and 4 of Table 1 (and Fig. 4), depending on the initial proportions of population sizes. In other words, whether the contact tracing is effective, or how effective the program is, might depend on the initial state of the epidemic in which a particular country or region is when intervention by contact tracing is first being implemented. This has important public health implications as it has been suggested in [4] that with antiretroviral therapy being more widely available, intervention policy based on intensive HIV testing and tracing of partners could be considered as a possible policy to control HIV/AIDS epidemics in other countries (also see [27]). Although we note that due to our assumption of low HIV prevalence and simulations using an initial proportion of undiagnosed infectives at 30% (or 70% detection rate of the HIV-positive individuals), the results might not be applicable to countries with low detection rate.

6. Finally, the linear force of infection term employed in this work can be considered as a linear approximation of the more commonly used standard incidence, *λ*XS/(S + X + Y_1 _+ Y_2 _+ Y_3_), when the total number of HIV-infectives, X + Y_1 _+ Y_2 _+ Y_3_, is small compared with S.

A stochastic Markov process model was proposed recently to describe the contact tracing detection in Cuba, in order to compare it with the usual random screening detection [28]. In this model, the component accounting for the contact-tracing feature is assumed to be valued in a space of point measures in order to take the time since detection into account. When an individual is identified as infected, it may contribute to detecting other infectious individuals by providing information related to persons with whom they have had possibly infectious contacts. This was expected to reinforce standard random-screening. Three models with three forms of the detection by contact-tracing have been considered by [28], where statistical estimation of the two rates of detection (by random detection and by contact tracing) using Cuban HIV/AIDS data shows that whatever the model used, the contact tracing detection reaches an efficiency equivalent to that of random screening detection approximately four years after the beginning of the epidemic. This fact, which had not been noticed by epidemiologists and health practitioners, is noteworthy since it goes countercurrent against the classical view on contact-tracing that it is efficient at the very beginning of its implementation and then less and less useful, insofar as the information it provides becomes redundant and obsolete as the contact tracing within a sexual network continues. More recently, approximate Bayesian computation is also applied to the Cuban HIV/AIDS data with contact-tracing and unobserved infectious population to make further observations concerning the efficiency of HIV detection system in Cuba [29].

Our results show that even if the secondary level of contact tracing is less effective in detection, with a substantial plunge in the percentage of detection from the first level contact tracing as noted previously, it still plays an important role in determining whether the disease can eventually be eradicated. This conclusion corroborates with the above conclusion by [28] on the effectiveness of contact tracing even a period of time after its implementation and even when the information it provides might become redundant and obsolete as we continue through the tertiary stages of contact tracing. Our results further indicate that contact tracing for HIV could be suitably used to remedy inadequacies in a universal testing program and in design of timely and effective intervention. Perhaps some combination of periodic (universal) random screening combined with contact tracing would be the most ideal and effective program to achieve the goal of quick detection and to increase early treatment, in order to attain eventual elimination [27].

As a final remark, the Family Doctor Program in Cuba, introduced in 1984, also provides a strong community-oriented primary care network which contributes to improving population health overall [30] and to respond to emerging and re-emerging diseases (including HIV), which also provide a frontline for prevention efforts. The family doctors' offices and community polyclinics play a pivotal role in prevention by providing counseling services, active screening services, promoting safer sex practices, and distributing educational materials, condoms, and lubricants, while also serving as the primary care providers for people with HIV participating in the ambulatory care system. Taken together, these initiatives profoundly affected how HIV manifested in Cuba [31] where in recent years the proportion of detections by contact tracing has fallen to 15% of those detected and some persons were detected through the family doctors before they could be traced by the contact tracing system. This is due to the fact that the number of reported contacts per detection has remained on the same level as before but as detection has almost doubled, the system could not effectively and swiftly cope with the larger number of contacts. Moreover, the family doctors are not really a random search or screening, since typically the family doctors counsel someone to test for HIV because that they suspect he/she might be infected. Therefore this element of detection is not readily modeled in our present model. In [28], it is found that a screening through contact tracing could reach peak efficiency after 4 years, and this drop-off conceivably could also apply to the family doctors program, as it is similarly not a random search. Perhaps a worthwhile future model extension, requiring detailed and updated data on the detection through the family doctor program, would include such consideration for understanding the detection and surveillance of HIV.

## Competing interests

The authors declare that they have no competing interests.

## Authors' contributions

YHH conceived and coordinated the study, construct the model, carried out the analysis, and wrote the first draft of the manuscript. YSW participated in the construction and analysis of the model, and in the writing of the manuscript. HdeA provided the data and participated in the analysis and the writing of the manuscript. RL participated in the analysis and the writing of the manuscript. All authors read and approved the final manuscript.

## Pre-publication history

The pre-publication history for this paper can be accessed here:

http://www.biomedcentral.com/1471-2334/10/194/prepub
